# The validity of diagnoses of non-affective psychotic disorder including schizophrenia in Swedish registers revisited – are the diagnoses valid for migrants and Swedish-born?

**DOI:** 10.1186/s12888-025-07282-5

**Published:** 2025-08-28

**Authors:** Jens Falk, Lidija Pavlovic, Susanne Wicks, Christina Dalman, Anna-Clara Hollander

**Affiliations:** 1 Epidemiology of Psychiatric Conditions, Substance Use and Social Environment (EPiCSS), Department of Global Public Health, Karolinska Institutet, Solnavägen 1E, Stockholm, Sweden; 2 Centre for Epidemiology and Community Medicine, Stockholm, Region Stockholm Sweden

**Keywords:** Non-affective psychotic disorder, Schizophrenia, Diagnostic validity, Register-based epidemiology, ICD-10, DSM-5

## Abstract

**Background:**

The first aim of the study was to assess the validity of non-affective psychosis diagnoses, including schizophrenia, for migrants and Swedish-born to determine if the registered diagnoses were of sufficient quality for epidemiological research. If the validity was insufficient, the second aim was to find out what the non-valid cases have in common to see if there was a feasible way to handle these cases in future studies.

**Study design:**

We validated the register-diagnoses of 179 randomly selected patients aged 18-48 living in municipalities with a high proportion of migrants, diagnosed with non-affective psychotic disorder (F20-F29 according to ICD-10), drawn from the Region of Stockholm’s medical records database by comparing them to their case notes to see if they fulfilled the DSM-5 criteria.

**Results:**

We found acceptable validity for non-affective psychotic disorder for migrant men (70.5%), low for Swedish-born men (60.0%), and even lower for women (50.0% for Swedish-born and 40.0% for migrants). There was no statistically significant difference between Swedish-born and migrants. The case notes revealed that by excluding cases with an additional diagnosis equivalent of psychotic disorder due to psychoactive substance (ICD10: F11X.5 and F11X.7) the validity was good for both Swedish-born and migrant men.

**Conclusions:**

This study supports continued use of the register-diagnoses but only after taking appropriate measures to avoid that patients with additional psychotic disorder due to psychoactive substance are not violating the validity. It also suggests caution when studying non-affective psychosis diagnoses among migrant women as the validity is low, possibly due to difficulties in separating non-affective psychosis from symptoms of other disorders with psychotic features.

## Introduction

Already in 1932, Ødegård reported that the prevalence of psychosis among Norwegian immigrants to the US was twice as high as that of Norwegians in Norway and native-born Americans [1]. Migration is one of the strongest and most consistently shown risk factors of schizophrenia and other non-affective psychoses [2–4]. The magnitude of this risk varies by migrant group and setting in which the study is conducted and follows migration flows [5–8]. The elevated psychosis rates in migrants have at times been met with scepticism based on suspected misdiagnosis among migrants. The possibility of the diagnoses of schizophrenia and other non-affective psychoses being due to misdiagnosis has thus been tested. In the Netherlands, a study tried out a culturally sensitive diagnostic tool in addition to standard diagnostic assessments [9]. When using the tool, the relative risk of schizophrenia observed in Moroccan migrants was reduced compared with the majority white Dutch population [9]. Still the overall levels of non-schizophrenia psychotic disorders remained over four times greater in Moroccan migrants, making it unlikely that these raised rates could be attributable to misdiagnosis only. In another test of misdiagnosing in the UK, the operationalized diagnoses were made by a panel of psychiatrists from over 13 different ethnic backgrounds and still found elevated rates of psychotic disorder in black and minority ethnic groups [10]. Still, cross-cultural diagnosing is difficult, hence additional tests of the validity of psychosis diagnoses among migrants are needed [9]. [10]

Visits to psychiatric care in Scandinavia and Finland are recorded in local and national administrative registers covering the entire population. Such registers have benefited schizophrenia and psychosis research immensely [11]. When using administrative registers created for generic purposes in research it is crucial to assess the quality of the information they contain, i.e. testing the diagnostic validity [12]. In this context diagnostic validity refers to if the recorded diagnoses meet the relevant diagnostic criteria measured as proportions of true positives compared with medical records (MR). The diagnosis is considered valid if it corresponds to the diagnostic criteria according to either *the International Classification of Diseases*(ICD) [13] or *the Diagnostic and Statistical Manual of Mental Disorders* (DSM) [14]. Schizophrenia (ICD-10 F20) in the Swedish registers has been validated 7 times between the year 1999 and 2010 [12]. [15, 16] Since 2010, there have not been any new validations made despite numerous changes in psychiatric care in Sweden. The changes a shift from a high level of inpatient care to more outpatient care. There has also been an increased migration to Sweden peaking in 2015 [17]. Relative to its population size, Sweden is now one of the European countries which have received the greatest number of migrants. Validation studies of schizophrenia in the Swedish administrative registers have found a validity between 68% and 95.5% ^12 16^ with the lower validity found in a study including not only the schizophrenia diagnosis but also schizoaffective disorder (F25) [16]. Despite often explored in research no study has so far tested the validity of the wider category of non-affective psychotic disorder F20-F29. Also, the validity of schizophrenia has not been tested with regard to migrants.

The first aim of the study is to assess the validity of non-affective psychosis diagnoses, including schizophrenia, for migrants and Swedish-born to determine if the registered diagnoses are of sufficient quality for epidemiological research. If the validity is insufficient, the second aim to find out what the non-valid cases have in common to see if there is a feasible way to handle these cases in future studies.

## Methods

### The setting, design and data source:

The validation aimed to examine whether patients with register diagnoses (first or second diagnosis) of non-affective psychotic disorder (F20-F29) according to ICD-10) [13] fulfilled the DSM-5 criteria according to the case notes in their medical records (MR)s.

F20-F29 include:

- F20 Schizophrenia.
- F21 Schizotypal disorder.
- F22 Persistent delusional disorders.
- F23 Acute and transient psychotic disorders.
- F24 Induced delusional disorder.
- F25 Schizoaffective disorders.
- F28 Other nonorganic psychotic disorders.
- F29 Unspecified nonorganic psychosis.

In Sweden, medical doctors use the DSM 5 for diagnosing, but register the diagnosis with ICD 10. As the medical doctors have used the DSM 5 for diagnosing, we choose to prepare a protocol based on DSM 5 to systematically document the symptoms and signs of each patient to judge whether the diagnostic criteria of non-affective psychotic disorder or schizophrenia were met. The DSM system was also chosen since it has become the global standard in psychiatric research and contains specific criteria for each diagnosis. If there was a disagreement between DSM 5 and ICD-10 the reviewing doctors used DSM 5. MR records were retrieved digitally from local specialist psychiatric treatment centres within the Region of Stockholm in Sweden. The MRs of the patients (see under Population and randomisation) were retrieved from the Region of Stockholm’s electronic MR database *Take Care. Take Care* was introduced within the Region of Stockholm in 2008 and used by most clinics by the year 2010. Twenty-two MRs were not possible to retrieve due to, for instance, the hidden identity of the MR holder. These MRs were deducted from the total number of MRs. The size of the medical records ranged from 1 to 100 pages, sometimes from different psychiatric caregivers.

### Population and randomisation

Eligible patients were those 18–48 years of age, with a physical visit or admission with a registered diagnosis (first or second) of non-affective psychotic disorder 2013–2016, living in two municipalities with a high proportion of migrants. Of these (789 in total), a random sample of 179 patients was selected. The SAS software package, Version 9.4, (SAS Institute Inc., Cary, NC, USA) was used in all statistical analyses and in drawing the random sample. As we selected persons living in an area with a high proportion of migrants, it was an oversampling of migrants as compared to a possible random sample of Sweden/Stockholm. This oversampling of migrants is the reason we are not presenting any overall estimates of the total validity of the study population. The age limit was chosen to limit the number of unretrievable MRs among older patients.

### Validation procedure

In Stockholm, treatment in adult psychiatric care for adults starts with a basic psychiatric examination (Basutredning in Swedish). If needed, this could be followed by an extended examination. A basic psychiatric examination is a comprehensive process to gather information about a patient’s mental health concerns and identify potential diagnoses. A basic psychiatric examination typically involves structured interviews with the patient and family members, standardized evaluation instruments such as the mandatory Alcohol Use Disorders Identification Test - Concise (AUDIT-C), Patient Health Questionnaire-9 (PHQ-9), Adult ADHD Self-Report Scale (ASRS-screening), EuroQol 5 Dimensions (EQ-5D), Clinical Global Impression – Severity (CGI-S), Mini International Neuropsychiatric Interview (M.I.N.I.) or Structured Clinical Interview for DSM-IV Axis I Disorders (SCID-I) and examination of any relevant medical or psychiatric records. When medical doctors are writing the MRs they write what is considered of value for planned treatment and this includes reporting the results of the evaluation instruments. The medical doctors do not have to verify operational criteria for each disorder.

The protocol of this study was prepared to systematically document all the symptoms and signs in the MRs, and more importantly, to document indications that other diagnoses would be more appropriate (i.e. the diagnose of non-affective psychosis or schizophrenia being a false positive). This was regardless of whether it was the first or secondary diagnosis in the register. The protocol was also used to record gender, age, interpreter/non-interpreter and use of alcohol or illicit substances. Two medical doctors (from now on called the reviewing doctors), in their psychiatric specialty training, reviewed five MRs together and twenty were double-checked by both separately to cross-validate their judgements to calculate the degree of agreement. We calculated the degree of agreement between the two doctors, in per cent and validity as proportions of true positives among the register-diagnoses. The differences between subgroups were compared using chi-square tests and Fisher’s exact test. As everyone in the population had a first- or second-registered diagnosis of non-affective psychotic disorder the diagnosis was not blind to the reviewing doctors.

### Subgroups

After the validation was finished, we linked the dataset with data from Statistics Sweden regarding migration status (born in Sweden or not) and gender.

### Identification of non-valid cases

For the invalid cases, the medical doctors reading the MRs documented the reason why the cases were considered invalid. To determine how to use register-diagnoses of non-affective psychotic disorder with the best possible validity in future studies, we searched the protocol described under “The validation process” for what features were common among the false-positive cases.

### Ethical approval

The study was approved by the Regional Ethical Review Board in Stockholm (Dnr 2016/1537-32).

## Results

Because of either protected identity, inaccessible MRs, or the death of the patient, some MRs could not be retrieved (28 persons out of the 179 selected, leaving 151 patients). The final sample included more men than women, and more Swedish-born than migrants, see Table 1. Among the migrants in the population, 61% moved to Sweden when they were 18 years or older, 39% were thus younger than 18 at immigration, and half of them were between 0 and 9 years old at immigration. The agreement between the two medical doctors validating the MRs was 95% i.e., out of 20 randomly selected cases 19 were judged the same by both.

**Table 1:** Demographic description of the retrieved cases of non-affective psychosis from the register

		*N* (%)
Total number of selected cases		179 (100%)
Number of inaccessible records		28 (16%)
Number validated		151 (84%)
Demographics of the final sample		
Gender according to MR	**Men**	99 (66%)
	**Women**	52 (34%)
Migration status according to Statistics Sweden	**Swedish born**	87 (58%)
	**Migrants**	64 (42%)

Among Swedish-born men, there were 55 validated register-cases (i.e. compared with their medical records) of non-affective psychosis diagnosis (F20-29) and 44 of these were true positive according to the reviewing doctors, i.e., they met DSM-5 criteria for the diagnosis, meaning 60.2% were considered valid, see Table 2. For schizophrenia (F20) there were 16 validated cases and 12 true positive meaning 75% were considered valid, see Table 2. Among migrant men there were 44 validated register-cases of non-affective psychosis diagnosis and 31 were true positive meaning 70.5% were valid, see Table 2. For schizophrenia there were 14 validated cases and 8 true positive meaning 57.1% were considered valid, see Table 2. The differences between Swedish born and migrant men were not statistically significant neither for non-affective psychotic disorder nor schizophrenia. For Swedish born women there were 32 non-affective psychosis diagnosis cases validated and 16 were true positive meaning 50.5% were considered valid, see Table 2. For schizophrenia there were 9 validated cases and 5 true positive meaning 55.6% were considered valid, see Table 2. For migrant women there were 20 non-affective psychosis diagnosis cases validated and 8 were true positive meaning 40.0% were considered valid. For schizophrenia there were 6 validated cases and 2 were true positive meaning 33.3% were considered valid, see Table 2. There was no difference in validity for male and female Swedish born or male of female migrants if the non-affective psychosis diagnose was registered at one or more instances. There were no statistical differences among those migrants who, according to the MR, had been assisted by an interpreter (interpreter 65% valid/no interpreter, 57% valid, *p* = 0.49)).

**Table 2:** Validity of the non-affective psychotic diagnoses and schizophrenia diagnoses in the register among the 151 mrs, n and (%), p-value for χ2 test of differences of proportions between the Swedish born and migrants

	Non-affective psychosis diagnosis F20-29			Schizophrenia F20		
	Swedish born	Migrant	Chi^2^-test	Swedish born	Migrant	Chi^2^-test/Fisher
Males and females						
n	87	64		25	20	
True positive n	49	39		17	10	
False positive n	38	25		8	10	
Proportion true positive	56.3%	60.9%	*p* = 0.57	68.0%	50.0%	*p* = 0.22
Males						
n	55	44		16	14	
True positive n	33	31		12	8	
False positive n	22	13		4	6	
Proportion true positive	60.0%	70.5%	*p* = 0.28	75.0%	57.1%	*p* = 0.30^#^
Females						
n	32	20		9	6	
True positive n	16	8		5	2	
False positive n	16	12		4	4	
Proportion true positive	50.0%	40.0%	*p* = 0.48	55.6%	33.3%	*p* = 0.61^#^
Birth year 1969–1977						
n	30	34		10	13	
True positive n	19	22		8	7	
False positive n	11	12		2	6	
Proportion true positive	63.3%	64.7%	*p* = 0.91	80.0%	53.8%	*p* = 0.38^#^
Birth year 1978–1986						
n	33	19		9	5	
True positive n	20	11		6	2	
False positive n	13	8		3	3	
Proportion true positive	60.6%	57.9%	*p* = 0.85	66.7%	40.0%	*p* = 0.58^#^
Birth year 1987–1995						
n	24	11		6	2	
True positive n	10	6		3	1	
False positive n	14	5		3	1	
Proportion true positive	41.7%	54.6%	*p* = 0.48	50.0%	50.0%	*p* = 1.00^#^

^#^ Fishers exact test

According to the MR-analysis made by the reviewing doctors 68.2% of the invalid cases among Swedish-born men and 41.7% among migrant men would more accurately have been diagnosed as the equivalent of psychotic disorder due to psychoactive substance (ICD10: F11X.5 and F11X.7). Thus, we excluded the patient with an additional psychotic disorder due to psychoactive substance use (F1X.5 and 7) according to MR (*n* = 32). This sample included 119 patients, see Table 3. When excluding patients with an additional psychotic disorder due to psychoactive substance use (F1X.5 and F1X.7) according to MR the validity among Swedish born men and migrant men increased (to 81.1% for non-affective psychosis diagnoses) but still low for Swedish born women and migrant women (57.1% for Swedish born women and 47.1% for migrant women for non-affective psychosis diagnoses).

**Table 3:** Validity of the non-affective psychotic diagnoses and schizophrenia diagnoses in the register among the 119 MRs after exclusion of those with additional psychotic disorder due to psychoactive substance use (F1X.5 and 7) according to MR, n and (%), p-value for χ2/Fishers exact test of differences of proportions between the Swedish born and migrants

	Non-affective psychosis diagnosis (F20-29)			Schizophrenia (F20)		
	Swedish born	Migrant	Chi^2^-test/ Fisher	Swedish born	Migrant	Chi^2^-test/ Fisher
Males and females						
n	65	54		21	17	
True positive n	46	38		17	10	
False positive n	19	16		4	7	
Proportion true positive	70.8%	70.4%	*p* = 0.96	81.0%	58.8%	*p* = 0.17^#^
Males						
n	37	37		14	12	
True positive n	30	30		12	8	
False positive n	7	7		2	4	
Proportion true positive	81.1%	81.1%	*p* = 1.00	85.7%	66.7%	*p* = 0.37^#^
Females						
n	28	17		7	5	
True positive n	16	8		5	2	
False positive n	12	9		2	3	
Proportion true positive	57.1%	47.1%	*p* = 0.51	71.4%	40.0%	*p* = 0.56^#^
Birth year 1969–1977						
n	27	30		8	12	
True positive n	19	21		8	7	
False positive n	8	9		0	5	
Proportion true positive	70.4%	70.0%	*p* = 0.98	100.0%	58.3%	*p* = 0.05^#^
Birth year 1978–1986						
N	25	17		7	4	
True positive n	19	11		6	2	
False positive n	6	6		1	2	
Proportion true positive	76.0%	64.7%	*p* = 0.50#	85.7%	50.0%	*p* = 0.49^#^
Birth year 1987–1995						
N	13	7		6	1	
True positive n	8	6		3	1	
False positive n	5	1		3	0	
Proportion true positive	61.5%	85.7%	*p* = 0.35#	50.0%	100.0%	*p* = 1.00^#^

^#^ Fishers exact test

According to the MRs common viable alternative diagnoses among women were bipolar disorder with psychotic symptoms. For migrant women a substantial part fulfilled a diagnosis of recurrent depressive disorder with psychotic symptoms, and/or post-traumatic stress disorder (PTSD) often with dissociative features.

## Discussion

 The first aim of the study was to assess the validity of non-affective psychosis diagnoses including schizophrenia, for migrants and Swedish born in a regional register, according to DSM-5 criteria, to determine if the register diagnoses are of sufficient quality for epidemiological research. We found that the validity of non-affective psychosis diagnoses in registers is low for both Swedish and migrant men and even lower for women. There were no statistically significant differences between Swedish born and migrants. The second aim was to find out what the invalid cases had in common according to the MRs to suggest how these should be handled in future studies. Overall, disorders due to psychoactive substance use were common. When excluding these cases, the validity reached acceptable levels for men but was still low among women. Prominent alternative diagnoses among the female were bipolar disorder with psychotic symptoms, recurrent depressive disorder with psychotic symptoms, and/or post-traumatic stress disorder.

This study supports the continued use of non-affective psychotic disorders including schizophrenia in register-based studies. However, only after excluding patients with additional psychotic disorder due to psychoactive substance, or possibly including these patients but using a sensitivity analysis to make sure the interference is not driven by the symptoms of these patients. There were no differences in validity with regards to migrants and non-migrants but differences between migrant men and women. This study underlines the need of caution when interpreting studies of non-affective psychotic disorders among migrant women as the validity could be lower in this group. This is likely due to difficulties in separating symptoms of non-affective psychotic disorder from other diagnoses with psychotic or dissociative features. This is in line with a study from our group that found that migrant status affected the risk of schizophrenia, schizoaffective disorder, affective psychotic disorders, and other non-affective psychotic disorders, but not non-psychotic bipolar disorder [18]. Affective psychotic disorders are so far not validated specifically among migrants in Sweden. PTSD is validated in the registers in 2019 [19] and has good validity in both migrant women and men, and the validity of PTSD was also high among migrants with psychotic symptoms, despite that psychosis and PTSD have similarities in terms of symptoms [19]. 

Schizophrenia diagnoses in the Swedish health care registers have been validated 7 times between 1987 and 2010 [12] and shown high accuracy except for a lower validity in a study by Reutfors (2010) [16]. Since then, there have been several changes potentially influencing the validity of the diagnoses. In the 1990 s many schizophrenia patients were still treated within inpatient care whereas nowadays a substantial part is treated in outpatient care only. Since 2006 psychiatric outpatient care are included in the national registers. Yet another difference since the last validity studies is the change from DSM-IV to DSM 5 with small changes of the primary criteria of the schizophrenia diagnosis.

A strength of the study is that the sample of persons with a non-affective psychotic disorder diagnosis was randomly selected from the two municipalises in the Stockholm Region representing two major psychiatric treatment centres with an almost complete coverage and this limits sampling bias. Another strength is that the doctors reviewing the MRs had a high agreement. A limiting factor with the method of validating diagnosis by reviewing MRs is a risk of misinterpretation when reading the case notes. For a DSM-criteria to be counted as fulfilled, the criteria needed to be explicitly mentioned in the MR. Sometimes symptoms may not have been noted in the MRs resulting in inflated numbers of false negatives, However, the medical doctors who validated the diagnoses read MRs from 2010 (at the latest) and up to 2016 thus basing their assessments on many years of assessments in the MRs. As we are not comparing patients with and without a registered diagnosis of F20-F29 diagnoses another limitation of this and other validity studies is that we know little of persons with an F20-F29 diagnosis not included in the registers. The ultimate gold standard of validation is always semi-structured diagnostic interviews [20]; however, using MRs is a standard method as the data is more easily available, more cost effective, and less intrusive for patients. An additional limitation is the small sample sizes, especially for women, thus some findings of low validity could be due to a type 2 error.

## Conclusions

This study supports the continues use the schizophrenia and non-affective disorders in register-register-based studies in Sweden after excluding or using a sensitivity analysis to make sure patient with additional psychotic disorder due to psychoactive substance are not violating validity. However, this study also underlines the caution when studying non-affective disorders among migrant women as the validity is low in this group, possibly due to difficulties in separating symptoms of non-affective psychosis and other disorders with psychotic or dissociative features.

## Data Availability

No datasets were generated or analysed during the current study.

## Abbreviations

DSM: Diagnostic and Statistical Manual of Mental Disorders
ICD: International Classification of Diseases
MR: Medical records
NAP: Non-affective psychotic disorder
PTSD: Posttraumatic stress disorder
